# Inhibition of Cellular Adhesion by Immunological Targeting of Osteopontin Neoepitopes Generated through Matrix Metalloproteinase and Thrombin Cleavage

**DOI:** 10.1371/journal.pone.0148333

**Published:** 2016-02-03

**Authors:** Alexander Jürets, Marie Le Bras, Günther Staffler, Gesine Stein, Lukas Leitner, Angelika Neuhofer, Matteo Tardelli, Edvin Turkof, Maximilian Zeyda, Thomas M. Stulnig

**Affiliations:** 1 Christian Doppler Laboratory for Cardio-Metabolic Immunotherapy and Clinical Division of Endocrinology and Metabolism, Department of Medicine III, Medical University of Vienna, Vienna, Austria; 2 AFFiRiS AG, Vienna, Austria; 3 Department of Plastic and Reconstructive Surgery, Medical University of Vienna, Vienna, Austria; The Forsyth Institute, UNITED STATES

## Abstract

Osteopontin (OPN), a secreted protein involved in inflammatory processes and cancer, induces cell adhesion, migration, and activation of inflammatory pathways in various cell types. Cells bind OPN via integrins at a canonical RGD region in the full length form as well as to a contiguous cryptic site that some have shown is unmasked upon thrombin or matrix metalloproteinase cleavage. Thus, the adhesive capacity of osteopontin is enhanced by proteolytic cleavage that may occur in inflammatory conditions such as obesity, atherosclerosis, rheumatoid arthritis, tumor growth and metastasis. Our aim was to inhibit cellular adhesion to recombinant truncated proteins that correspond to the N-terminal cleavage products of thrombin- or matrix metalloproteinase-cleaved OPN *in vitro*. We specifically targeted the cryptic integrin binding site with monoclonal antibodies and antisera induced by peptide immunization of mice. HEK 293 cells adhered markedly stronger to truncated OPN proteins than to full length OPN. Without affecting cell binding to the full length form, the raised monoclonal antibodies specifically impeded cellular adhesion to the OPN fragments. Moreover, we show that the peptides used for immunization were able to induce antisera, which impeded adhesion either to all OPN forms, including the full-length form, or selectively to the corresponding truncated recombinant proteins. In conclusion, we developed immunological tools to selectively target functional properties of protease-cleaved OPN forms, which could find applications in treatment and prevention of various inflammatory diseases and cancers.

## Introduction

Osteopontin (OPN), also known as secreted phosphoprotein 1, is encoded by the gene *SPP1* and is a member of the small integrin-binding ligand N-linked glycoprotein (SIBLING) family [1]. OPN is secreted into the body fluids such as milk, urine, and blood, but it is also part of the extracellular matrix of many tissues [2, 3]. Overexpression of OPN is linked to cancer, rheumatoid arthritis, atherosclerosis, and obesity-induced adipose tissue (AT) inflammation [4–7], in which it represents one of the most strongly overexpressed cytokines [8]. Our group showed that neutralizing osteopontin with polyclonal antibodies reduces AT inflammation and insulin resistance in a diet-induced obesity mouse model [9].

OPN promotes cell migration, adhesion, and activation of T lymphocytes and macrophages via interaction with integrins and multiple variants of CD44 [10]. Within the central region of OPN, integrins can bind two described binding motifs. The integrins α_v_β_1_, α_v_β_3,_ α_v_β_5_, α_v_β_6_, and α_5_β_1_ bind a canonical RGD binding motif, which is ubiquitous on extracellular matrix proteins. Cleavage of OPN after Gly^166^ or Arg^168^ of the adjacent SVVYGLR motif by the proteolytic enzymes matrix metalloproteinase (MMP) or thrombin to obtain mOPN or tOPN increases the adhesion via the RGD binding α_v_β_3_ and α_5_β_1_ through increased accessibility. Furthermore, cleavage by thrombin is necessary in order to be bound by the integrin α_9_ [11–15]. Thus, in conditions with increased thrombin or MMP activity, such as obesity-induced AT inflammation [16–18], atherosclerosis [19, 20], rheumatoid arthritis [21], asthma [22, 23], and cancer [24], OPN-neoepitopes with increased adhesive properties are generated. Targeting neoepitopes, which are generated and increased in pathological conditions, may provide interesting avenues for immunological approaches that aim at neutralization of an endogenous protein with multiple functions, such as OPN, while minimizing adverse effects.

In this study we investigated whether OPN fragments can be specifically blocked without affecting the function of the full-length form. Since there is a lack of specific and functional active antibodies against the MMP-cleaved form of OPN, we created new monoclonal antibodies and assessed their ability to block adhesion of HEK 293 cells to recombinant OPN fragments, without affecting binding to full length OPN (flOPN). In addition, we investigate an active immunization approach to specifically target the human MMP- or thrombin cleaved OPN form with murine post immune sera in order to functionally block adhesion of a human cell line.

## Methods

### Ethics statement

This study was conducted according to the principles expressed in the Declaration of Helsinki and Good Clinical Practice Guidelines at the Department of Medicine III, Medical University of Vienna, and has been previously approved by the Ethics committee of the Medical University of Vienna (EK no. 275/2006 and 290/2006). All Patients provided written informed consent to be included in the studies.

For animal experiments this study was approved by the Committee on the Ethics of Animal Experiment of the Medical University of Vienna and the Austrian Federal Ministry for Science and Research (Permit Number: BMWF-66.009/0096-II/10b/2008). Diet and housing were guideline conform according to the European Convention for Protection of Vertebrate Animals Used for Experimental and Other Scientific Purposes. Animal experiments adhered to the 3 Rs of animal welfare (Replacement, Reduction and Refinement).

### Isolation of human adipose tissue stromal vascular cells

Human subcutaneous AT was obtained by liposuction or elective abdominoplasty. Stromal vascular cells were isolated as previously described [25]. In summary, AT was homogenized and digested with collagenase type I (Worthington, Lakewood, NJ) at 37°C. Samples were filtered and the stromal vascular cell fraction was obtained by centrifugation. Red blood cells were removed by lysis, remaining cells washed in DPBS and subjected to flow cytometry.

### Flow cytometry

For flow cytometric analysis of OPN binding surface molecules, cells were detached using trypsin-EDTA solution (GIBCO, Life Technologies, Carlsbad, CA, USA) and labeled for 45 minutes on ice with directly labeled antibodies. Thereafter, cells were washed twice with ice cold 1x DPBS and analysis was performed on a BD FACSCantoII (BD Biosciences, Franklin Lakes, NJ, USA). Stromal vascular cells (SVCs) were gated for CD144^+^ endothelial cells, CD34^+^CD144^-^ preadipocytes, and CD45^+^ immune cells.

### Antibodies and reagents

We used the isotype control IgG1 (M5284, Sigma-Aldrich, St. Louis, MO, USA) as well as control serum (M5905, Sigma-Aldrich, St. Louis, MO, USA). The monoclonal mouse antibodies against tOPN and mOPN were generated in our lab. For mAb 9–3 we used GDSVVYG and for mAb 21–5 TYDGRGDSVVYG-CO-NH_2_ as immunization peptides. These antibodies were thoroughly analyzed using peptide-specific ELISAs as well as the Biacore technology (GE Healthcare, Little Chalfont, UK), which revealed GDSVVYG-COOH (carboxyl residue-specific) and SVVYG irrespective of a free carboxyl residue as the probable epitopes bound by mAb 9–3 and mAb 21–5, respectively, with high affinity and selectivity (data not shown). Antibodies for flow cytometry are shown in S1 Table.

### Expression vectors

**Fig 1:**
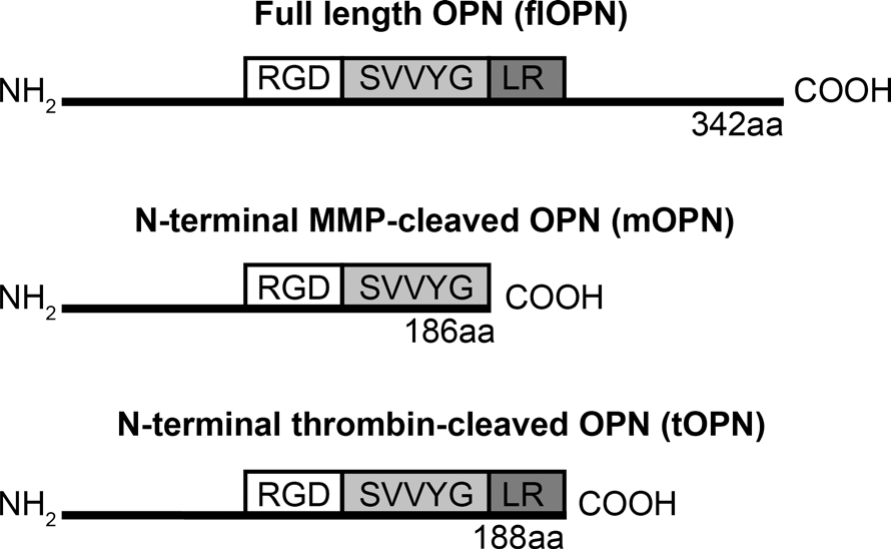
Recombinant OPN schemata. Schemata of used recombinant OPNs, which highlights the canonical integrin binding domain RGD as well as the contiguous cryptic integrin binding domain SVVYGLR. Depicted are the recombinant full length OPN form (flOPN) and OPN fragments, which mimic the MMP (mOPN) or thrombin (tOPN) cleaved N-terminal OPN. Furthermore, protein lengths in amino acids are shown. aa, amino acids.

The cDNA of human OPN without the signal peptide (aa 17–314) was obtained by PCR-amplification of MGC human SPP1 sequence-verified cDNA (Clone ID 3828885, Accession BC017387, Thermo Fisher Scientific, Waltham, MA, USA) using one forward primer (5’-AGCGGCTCTTCAATGATACCAGTTAAACAGGCTGATTC-3’) and depending on the construct 3 different reverse primer: flOPN (5’-TCACGTAGAAGACTCCAGTTACCCTCTTCTCGGCGA-3’, mOPN (5’-CCACTATCACACCAAATACCTATCCCCTCTTCTCGGCGA-3’) and tOPN (5’-CACACCAAATACCTGACTCCATCCCCTCTTCTCGGCGA-3’). The PCR product was inserted into a pENTRY-IBA-51 vector and the correct insertion was verified by sequencing. The gene of interest was transferred to StarGate Acceptor vector pCSG-IBA142 or pCSG-IBA144 following the StarGate manual (IBA, Göttingen, Germany). With the acceptor vectors a BM40 signal peptide as well as 6x histidine or Strep-tags were introduced, which resulted in secreted proteins with N- and C-terminal tagged flOPN, or N-terminal tagged mOPN and tOPN fragments (Fig 1).

### Cell culture

Human embryonic kidney cells HEK 293 (ATCC, Manassas, VA, USA) and the 293 c18 (ATCC, Manassas, VA, USA) descendent were cultured in DMEM (Sigma-Aldrich, St. Louis, MO, USA), supplemented with 10% FBS (PAA Laboratories, GE Healthcare, Little Chalfont, UK), L-glutamine (GIBCO, Life Technologies, Carlsbad, CA, USA), penicillin streptomycin (GIBCO, Life Technologies, Carlsbad, CA, USA), and, for 293 c18 cells only, 250 μg/ml geneticin sulfate (GERBU, Heidelberg, Germany).

### Expression and purification of recombinant OPN

293 c18 cells were transfected with Lipofectamine LTX (Invitrogen, Life Technologies, Carlsbad, CA, USA) according to manufacturer’s instructions. Medium was changed and collected every 2 to 3 days. Cell debris were removed by centrifugation at 500 x g for 10 min at 4°C, and pH was adjusted to pH 8.0. Supernatant from doubletagged flOPN or histidine tagged fragments was first applied to gravity flow columns packed with Ni-NTA His-bind Resin (Novagen, Merck KGaA, Darmstadt, Germany), columns were then washed with washing buffer (50 mM sodium phosphate, 300 mM NaCl, and 20 mM imidazole, pH 8.0), and bound proteins were eluted with elution buffer (50 mM sodium phosphate, 300 mM NaCl, and 250 mM imidazole, pH 8.0). Fractions containing flOPN, as well as unfractionated supernatants containing Strep-tagged OPN fragments were applied to gravity flow columns packed with Strep-Tactin sepharose (IBA, Göttingen, Germany). After washing with equilibration buffer (100 mM TrisCl, 150 mM NaCl, and 1mM EDTA, pH 8.0), bound proteins were eluted with elution buffer (100 mM TrisCl, 150 mM NaCl, 1 mM EDTA, and 2.5 mM D-desthiobiotin). Recombinant proteins were dialyzed against 1x DPBS using Slide-a-lyzer Dialysis Cassettes (Thermo Fisher Scientific, Waltham, MA, USA).

Protein concentration was determined with BCA protein assay (Pierce Biotechnology, Thermo Fisher Scientific, Waltham, MA, USA). Purified proteins were analyzed by 12% SDS-PAGE, followed by colloidal blue silver staining (0.12% coomassie brilliant blue G-250, 10% ammonium sulfate, 10% phosphoric acid, and 20% ethanol) [26]. Identity of OPN was confirmed by immunoblotting. To this end, proteins were transferred to a PVDF membrane by semi-dry transfer after SDS-PAGE. The membrane was probed with a primary polyclonal anti-OPN antibody (AF1433, R&D Systems, Minneapolis, MN, USA), secondary horseradish peroxidase-coupled donkey anti-goat IgG (abcam, Cambridge, MA, USA) and detected with BM chemiluminescence western blot substrate (Roche Applied Science, Penzberg, Germany) on a Fusion FX7 imager (Vilber Lourmat, Eberhardzell, Germany) (S1 Fig).

### Cell adhesion assay

HEK 293 cells were grown to 70–80% confluence, harvested with Versene (GIBCO, Life Technologies, Carlsbad, CA, USA) and resuspended in 10% FBS containing DMEM medium without phenol red. Cells were labeled for 30 minutes at 37°C with 1.5 μM cell tracker green CMFDA (Molecular Probes, Eugene, OR, USA). Labeled cells were washed once in PBS. Cell adhesion assay using V-well microtiter plates was adapted from the procedure described by Weetall *et al*.[27]. In detail, recombinant OPN or fragments thereof were immobilized on polystyrene 96 V-well microtiter plates (greiner bio-one, Kremsmünster, Austria) in 50 μl coating buffer (20 mM Tris and 15 mM NaCl, pH 9.4) for 1 h at 37°C or overnight at 4°C. Free binding sites were blocked by adding 100 μl 1% BSA in 1x DPBS for 1 h at 37°C. Plates were washed twice with 100 μl DPBS. For blocking antibodies or antisera diluted in 50 μl 1x DPBS were added and incubated for 1 h at 37°C. Plates were washed twice, and 20.000 fluorescent labeled cells in 50 μl DMEM with 10% FBS were added and incubated for 30 min at 37°C. For integrin inhibition the assays were performed by incubating the cells with 1 μM RGES peptide (Abbiotec, San Diego, CA, USA), cilengitide, or TF-14035 (both Medchemexpress, Princeton, NJ, USA) during the 30 minutes labeling and the 30 minutes incubation on the plates. Plates were centrifuged at 80 x g for 5 min and fluorescent cells in the nadir of the well were measured using an EnSpire Multimode Reader (Perkin Elmer, Waltham, MA, USA) at bottom reading mode, excitation 490 nm and emission 520 nm wavelengths.

Adhesion was determined as previously shown by Schack *et al*. by comparing the signal from the OPN-coated wells with the signal of blocked uncoated wells according to the formula: “*Adhesion [%] = 1—MFI*_*OPN*_*/ MFI*_*0*_”, where MFI_0_ is the mean fluorescence intensity (arbitrary units) without any prior coated protein and MFI_OPN_ at a given OPN concentration [28].

Furthermore, for testing for coating differences of the used OPN forms we performed a modified BCA protein Assay similar to Hui *et al*., where we coated the microplates with 10 μg/ml protein in triplicates and as control 6 wells without protein [29]. Plates were washed and 100 μl BCA working solution was incubated for 2 hours at 60°C. Absorbance was detected at 562 nm using an EnSpire Multimode Reader (Perkin Elmer, Waltham, MA, USA). The limit of detection was calculated at 1 μg/ml protein by using a BSA standard (S2 Fig).

### Antibody reactivity and mouse titer determination

Antibody binding and mouse titers were determined by standard ELISA techniques. Recombinant OPN forms were immobilized in duplicates on an ELISA plate (Corning, Corning, NY, USA). Binding antibodies were detected with HRP-labeled anti-mouse antibodies (Jackson ImmunoResearch, West Grove, PA, USA). ABTS was added and absorbance was measured at 405 nm and EC50 was calculated.

### Post immune sera

Mimetic peptides of the central integrin binding region (PTVDTYDGRGDS against flOPN, GDSVVYG against the C-terminus of the mOPN fragment, VVYGLR against the C-terminus of the tOPN fragment, and the scrambled control SGRVYGDLVGRD) were coupled with N-gamma-maleimidobutyryl-oxysuccinimide ester to keyhole limpet haemocyanin (KLH). Conjugates were mixed with Alhydrogel® (Brenntag, Mülheim, Germany) adjuvant. 30 μg peptides with 0.2% Alhydrogel® were applied in 200 μl to animals with each vaccination.

10–12 week old wild type C57BL/6 mice (Janvier Labs, Le Genest St. Isle, France) with 6 mice per group were vaccinated four times in a biweekly interval. Blood was taken at the beginning and before each vaccination. Thereafter, post immune sera were taken under isofluorane anesthesia through cardiac puncture. Afterwards, the mice were euthanized by cervical dislocation.

### Statistical analysis

Data are presented as mean ± standard error of the mean. Groups were compared using two-way ANOVA followed by Holm- Šídák’s post-hoc test, using GraphPad PRISM Version 6.04 software (GraphPad Software Inc., San Diego, CA, USA). A p-value below 0.05 was considered significant.

## Results

### Cell adhesion to OPN

**Fig 2:**
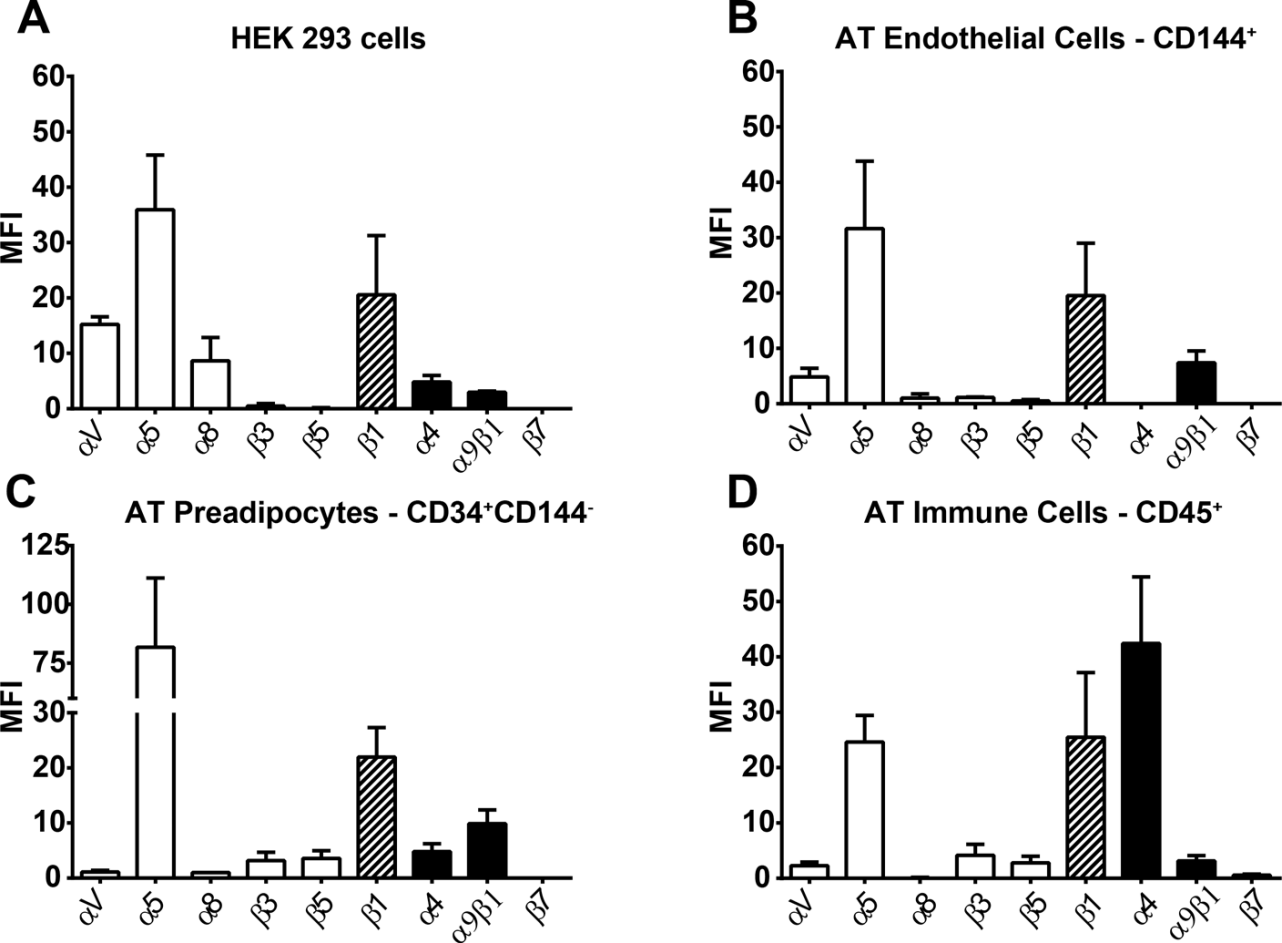
Surface profiling of OPN binding integrins. Quantitative flow cytometry analysis of OPN-binding integrins on the surface of HEK 293 cells (A) as well as on AT derived SVCs. Depicted are SVCs that were gated for CD144^+^ endothelial cells (B), CD34^+^CD144^-^ adipocyte precursor cells (C), and CD45^+^ immune cells (D). White bars represent RGD-dependent integrins, black bars SVVYG- or SVVYGLR-dependent integrins, and hatched bars integrins, which bind the whole central region. MFI (arbitrary units) is calculated by the MFI from the anti-integrin antibodies minus the MFI from the labeling control (control IgG-PE for α_4_, α_5_, α_8_, α_V_, α_9_β_1_, and control IgG-FITC for β_1_, β_3_, β_5_, β_7_, CD44, CD44v6) divided by the respective labeling control. Depicted are the means ± SEMs of at least 3 independent experiments. MFI, mean fluorescence intensity.

First, we compared HEK 293 cells and cells from the AT stromal vascular fraction concerning their expression of RGD-dependent α_V_, α_5,_ α_8_, β_3_, β_5_, and β_7_ integrin chains and those binding the cryptic domain, namely α_4_, and α_9_β_1_ integrins. Integrin β_1_ containing integrins–depending on the combined α chain–are able to bind both binding motifs and β_1_ expression is high in HEK 293 (Fig 2A), AT endothelial cells (Fig 2B), AT preadipocytes (Fig 2C), and AT immune cells (Fig 2D). Notably, also the RGD-dependent α_5_ integrin is highly expressed in all investigated cell types, which binds OPN stronger upon cleavage [14]. In contrast, only AT immune cells showed a high expression of the integrin α_4_. These data suggest that all investigated cell types are able to bind the RGD sequence and increase their binding strength upon proteolytic cleavage due to the high α_5_ expression. Of note, HEK 293 cells that express RGD- and SVVYGLR-binding integrins appear suitable for investigation of cellular adhesion to both, flOPN, to which only RGD-dependent integrins can bind, and the OPN fragments mOPN and tOPN that feature the unmasked SVVYG or SVVYGLR integrin binding sites, respectively.

**Fig 3:**
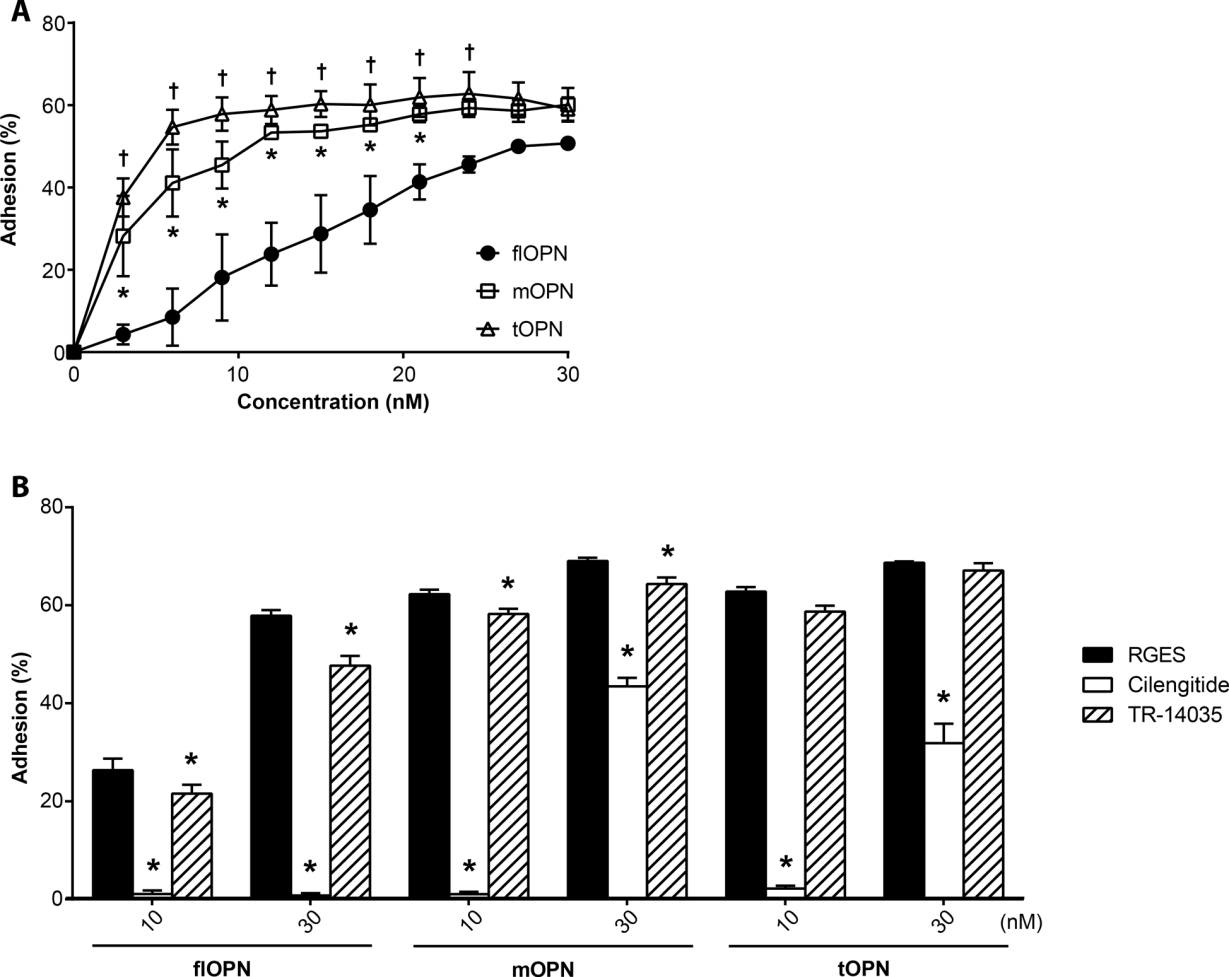
HEK 293 cell adhesion assays and blockade thereof with integrin inhibitors. **(A)** Cellular adhesion in percent of HEK 293 cells from 0 to 30 nM coated recombinant OPN forms in 3 nM steps. Depicted are the means ± SEM of 3 independent experiments. * and ^+^ indicate significance of cellular adhesion to mOPN or tOPN in comparison to full length OPN, respectively. (B) Blockade of cellular adhesion of HEK 293 cells at 10 or 30 nM coated recombinant OPN forms with 1μM antagonistic integrin inhibitors. RGES (black bars) was used as a control peptide. Cilengitide (white bars) inhibits the integrins α_V_β_3_, α_V_β_5_, and α_5_β_1_. TR-14035 (hatched bars) inhibits the integrins α_4_β_7_ and α_4_β_1._ Depicted are the means ± SEM of 3 independent experiments. * indicate significance of cell adhesion to the RGES control peptide.

Thus, we precoated microplates with flOPN or OPN fragments at concentrations from 0 to 30 nM, and studied HEK 293 cell adhesion to the proteins. As expected, cells adhered to significantly lower concentrations of both OPN fragments in comparison to the full length form (Fig 3A). Already 10 nM of OPN fragments induced maximal cell adhesion, whereas 30 nM of flOPN was necessary to induce cell adhesion to the same extend. Therefore, we decided to evaluate inhibition of HEK 293 at OPN concentrations of 10 nM and 30 nM.

To investigate which integrins are involved in the adhesion of HEK cells to the OPN forms, we used the antagonistic integrin inhibitors cilengitide, which inhibits the binding to the RGD motif of α_V_β_3_, α_V_β_5_, and α_5_β_1_ [30], and TR-14035, which inhibits the binding of integrins α_4_β_7_ and α_4_β_1_ (Fig 3B). In comparison to the control peptide RGES, both integrin inhibitors reduced the adhesion to flOPN and mOPN significantly, whereas only cilengitide reduced the adhesion to tOPN. However, in all instances cilengitide reduced the adhesion to a by far higher extent than TR-14035.

### Blockade of OPN-mediated cell adhesion with monoclonal antibodies

**Fig 4:**
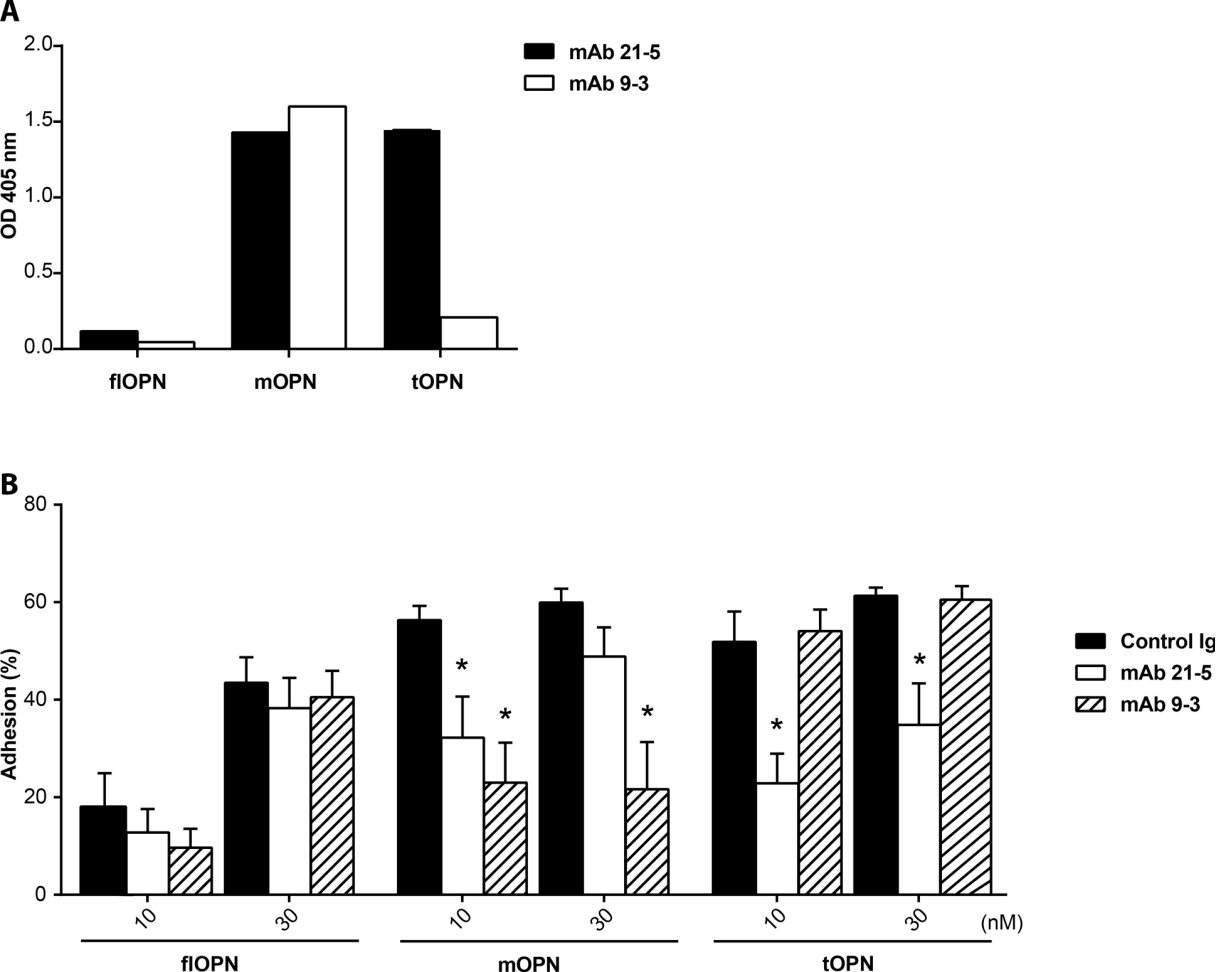
Selective targeting OPN fragments with antibodies. (A) ELISA data of mAb 21–5 (white bars) and mAb 9–3 (hatched bars) against flOPN, mOPN, and tOPN. OPN bound antibodies were detected with HRP-labeled anti-mouse antibodies. OD405 nm represents the ABTS substrate consumption after incubating 30 minutes in arbitrary units. Depicted are the means ± SD of duplicates of a repeated experiment. (B) Cellular adhesion in percent of HEK 293 cells at 10 and 30 nM coated recombinant OPN form. Plates were preincubated either with 5 μg/ml isotype control (black bars), mAb 21–5 (white bars) or mAb 9–3 (hatched bars) antibodies. Depicted are the means ± SEMs of 3 independent experiments with triplicates. * indicates significant reduction of cellular adhesion in comparison to the isotype control.

We designed the monoclonal antibodies mAb 21–5 and mAb 9–3, specific for the neoepitope of thrombin- and MMP-cleaved OPN, respectively. In contrast to mAb 9–3, which is specific for the mOPN fragment in ELISA, mAb 21–5 binds both cleaved forms (Fig 4A). Importantly, none of the antibodies recognize flOPN.

We further tested whether the binding of the antibodies to the neoepitopes impeded cell adhesion to the N-terminal fragments of cleaved OPN. The antibody mAb 21–5 targeting the SVVYGLR motif significantly reduced adhesion to tOPN at both concentrations 10 and 30 nM as well as to mOPN at a concentration of 10 nM (Fig 4B). In contrast, preincubation with mAb 9–3, which recognizes the C-terminus of mOPN, significantly reduced adhesion solely to mOPN.

Of note, after incubation of OPN with isotype control antibodies, the extent of cell adhesion was comparable to the adhesion observed without preincubation with any antibodies. Thus, in accordance with the binding properties (Fig 4A), mAb 21–5 impeded adhesion to both cleaved OPN forms (although more potent for tOPN), whereas mAb 9–3 was specific for mOPN regarding its inhibitory potential.

### Blockade of OPN-mediated cell adhesion with antisera

**Fig 5:**
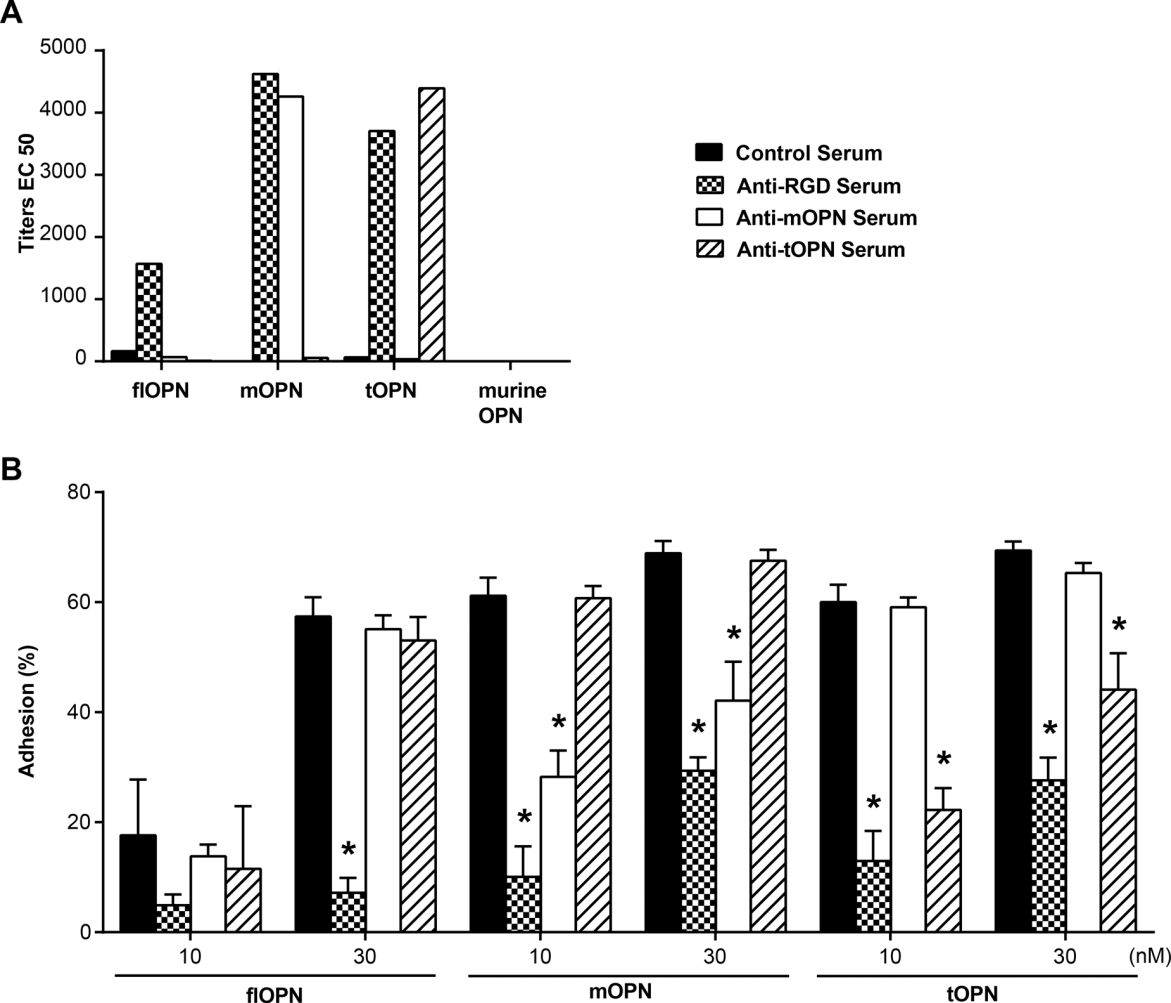
Selective targeting OPN fragments with post-immune sera. (A) ELISA data of antisera tested for reactivity against coated human flOPN, mOPN, tOPN, and recombinant murine flOPN. OPN bound mouse antibodies were detected with HRP-labeled anti-mouse antibodies. Titer represents half maximal effective concentration (EC50) was measured after incubating 30 minutes with ABTS substrate. Depicted are the means ± SD of duplicates of a repeated experiment. (B) Cellular adhesion in percent of HEK 293 cells at 10 and 30 nM coated recombinant OPN forms. Plates were preincubated with 1:50 diluted antisera either against a scrambled control peptide, the RGD motif, mOPN or tOPN. * indicates significant reduction of cellular adhesion in comparison to control serum against the scrambled peptide. Depicted are the means ± SEMs of 3 independent experiments.

In the next step, we aimed to test whether the functionality of antibodies induced by peptide vaccination *in vivo* was comparable to the functionality of our purified monoclonal antibodies. Therefore, mice were vaccinated with keyhole limpet haemocyanin (KLH)-coupled peptides mimicking the central integrin binding motif of thrombin- and MMP-cleaved OPN, the RGD within OPN, as well as an irrelevant control peptide. First, the specificity of the sera for the recombinant proteins was determined. Sera induced with RGD-containing peptide reacted against all three human OPN forms, whereas vaccination with the peptides corresponding to the neoepitopes resulted in antisera that were selectively reactive against the respective protein fragments (Fig 5A).

Next, we investigated the antisera for adhesion-impeding properties. The addition of control serum did not impede adhesion of cells to all forms of OPN (Fig 5B). In contrast, sera that targeted the RGD integrin binding motif significantly impeded cellular adhesion to flOPN at 30 nM and mOPN and tOPN at 10 nM and 30 nM. Strikingly, sera against the neoepitopes of mOPN and tOPN selectively impeded adhesion to their respective OPN fragment. In summary, the anti-RGD serum inhibited cellular adhesion to all tested OPN-forms, whereas anti-mOPN and anti-tOPN serum specifically blocked the target intended by our peptide design.

## Discussion

Immunotherapies against endogenous molecules are becoming a widely investigated strategy in the development of treatment for non-communicable diseases such as hypertension, atherosclerosis, rheumatoid arthritis, Alzheimer’s disease and type 1 diabetes mellitus [31–33]. Several immunotherapies targeting cytokines are approved for passive immunization. For instance, antibodies against TNFα are used for the treatment of rheumatoid arthritis, Crohn’s disease, and psoriasis [32]. Notably, other antibodies that inhibit the binding of PD-1 on T cells to its ligand PD-1L, which is expressed on some tumors, are used to treat cancer [34]. Therefore, we aim at immunologically targeting OPN, which is a player in a multitude of pathological conditions [21, 35, 36]. However, passive immunization is a cumbersome and expensive treatment, where high doses of antibodies have to be repeatedly applied over a prolonged period of time. In addition, due to the high doses of injected monoclonal antibodies, anti-antibodies may be induced, which may diminish the efficacy of the treatment. Active immunizations, in contrast, require only small amounts of recombinant protein or peptide vaccines to be injected and repeated only after prolonged intervals of time. Antibody titers are usually inferior to passive immunization but active immunization bears reduced risk to develop anti-antibodies though may result in other autoimmune effects [32].

Concerning the major integrin binding site of OPN, targeting of the RGD motif might lead to adverse effects since this sequence is canonically expressed in the extracellular matrix on ubiquitously expressed proteins such as vitronectin, fibronectin, collagens, thrombospondin, von Willebrand factor, and fibrinogen. Although other studies tried to target the α_V_ integrins in cancer and other diseases, they indirectly targeted RGD by blocking the integrins with antibodies or blocking their binding with soluble mimetic peptides [37]. If normal cells lose connection to the matrix, they will stop migration [38] or undergo apoptosis [39]. Adverse effects may be circumvented by addressing neoepitopes with increased abundance under pathological conditions. For instance, we previously showed that during AT inflammation cleaved OPN forms are more abundant in human AT [18] as also shown for atherosclerotic plaques and synovial fluids in rheumatoid arthritis [20, 40]. Others showed that antibodies against the neoepitopic mouse homologue OPN region SLAYGLR could reduce inflammation in rheumatoid arthritis [41] and reduce renal crystal formation [42]. The group of Uede even designed an antibody similar to our used mAb 21–5 which recognized the SVVYGLR of human OPN, which showed promising results in the treatment of rheumatoid arthritis in a primate study [43]. However, a phase 1 clinical study performed in humans with a high diseases activity (average DA28S of 6) was tolerated well, but did not show any disease improvements [44]. In contrast to our used antibodies, it is not known if this antibody also binds the N-terminal MMP-cleaved OPN fragment. We show for the first time that the antibodies mAb 9–3 and mAb 21–5 can block the adhesion of human cells to the human form of the N-terminal MMP-cleaved OPN, and mAb 21–5 also to thrombin-cleaved OPN, in an *in vitro* model.

HEK 293 cells adhere to flOPN by means of integrins α_V_ [45], α_8_ [46], and by the non-integrin surface molecule CD44 [47, 48] in particular its isoforms CD44v6 [49] and CD44v10 [50]. Since the CD44 binding sequence on OPN is insufficiently determined for targeting, we concentrated on integrins in this study. However, HEK 293 cells strongly increased adhesion upon thrombin or MMP cleavage of OPN, similar to different cell lines as described [11, 12, 14, 51]. The increased adhesion of HEK 293 cells might be due to the strong expression of integrin α_5_, which shows enhanced affinity to tOPN [14] as well as the moderate expression of α_4_ [52, 53]. Interestingly, Yokosaki *et al*. showed that adhesion dependent on the integrin α_5_β_1_ is only increased if OPN is cleaved by thrombin and not by MMP-3[14]. However, in our conditions and similar to other publications [11, 51] adhesion was higher to 10 nM mOPN than to flOPN, although integrin profile points at integrin α_5_β_1_ as the major OPN-binding integrin. The blockade of mOPN adhesion with cilengitide also suggests that in our experiments the increased binding to MMP-cleaved OPN is indeed integrin α_5_ dependent and probably due to binding to integrin α5β1, since β3 and β5 expression was hardly detectable on the cells used here. Other also showed that adhesion or cell attachment is enhanced after MMP cleavage of OPN [11, 51, 54]. This increased adhesion to the cleaved form seems to be dependent on the RGD binding integrins and integrin α_9_. Contrary to this enhanced adhesion upon proteolytic cleavage of osteopontin seems to be integrin α_4_ mediated. Integrin α_4_ adhesion of MnCl_2_-activated Jurkat cells is not influenced by cleavage or post-translational modification although it binds to SVVYG [29, 55]. However, antibody mAb 21–5 could not detect flOPN although it also binds SVVYG. A reason for this might be that although OPN is an intrinsically disordered protein the central region shows less conformational flexibility than the rest of the protein [56], which probably prevents binding of mAb 21–5 to the full form and is the reason for increased binding of RGD-dependent integrins upon cleavage. Probably for the same reason we observed that the anti-RGD serum showed a higher reactivity against cleaved forms than against flOPN in the ELISA.

In human AT we found that endothelial cells and preadipocytes express mainly the tOPN binding α_5_ and α_9_β_1_ [13, 14], which may, according to our data, bind mOPN as well, whereas immune cells indicated binding properties for all forms of OPN because of a high integrin α_4_ expression [53]. This difference may be utilized to specifically inhibit, for instance, the activation of immune cells by immunological targeting of proteolytically cleaved OPN forms, for which mAb 9–3 and mAb 21–5 showed promising properties. The HEK 293 cells used in adhesion assays expressed RGD-binding integrins to a higher extent than the investigated AT-derived cells, which expressed only α_5_ at a reasonable level. Therefore, the pro-inflammatory cell-activating abilities of OPN protease cleavage, especially by MMP, might be even underestimated when testing HEK 293 cells. We showed that HEK 293 adhesion is increased to the N-terminal MMP or thrombin cleaved OPN and that this is mainly due to RGD-dependent integrins. Blockade of the SVVYG binding α_4_ integrin also reduced adhesion to flOPN and mOPN, but not to tOPN which could be due to the relatively low expression of α_4_ integrins on the used cell line.

In this study we specifically inhibited cellular adhesion to integrin-binding neoepitopes on recombinant proteins that were mimicking the N-terminal fragments of MMP or thrombin-cleaved OPN with monoclonal antibodies or peptide vaccination-induced antisera. Thereby we were laying ground for passive and active immunization approaches to treat chronic inflammatory diseases or cancer, which have been shown to be driven by OPN. Both generated monoclonal antibodies where able to bind the cryptic integrin region without recognizing the full length form. mAb 9–3 was specific solely for mOPN, whereas mAb 21–5 antibodies bound both truncated forms. For further evaluation of an active immunization approach, we investigated the functionality of post-immune sera. Sera elicited by immunization with an RGD-containing peptide inhibited adhesion to all OPN forms of tested protein. Although it showed better reactivity against the peptides derived from cleaved forms, functionally it somewhat better blocked adhesion to the full length than the cleaved forms (particularly at 30 nM OPN, Fig 5B. Hence, reactivity against a peptide does not necessarily lead to functional blockade of the respective region in the protein [57]. Interestingly, with antisera and mAb 9–3 adhesion to mOPN was inhibited without interfering with adhesion to tOPN, even though mOPN is just 2 C-terminal amino acids shorter. Furthermore, adhesion solely to tOPN was also inhibited by antisera, whereas mAb 21–5 impeded adhesion to both truncated proteins.

In conclusion, we show for the first time that antibodies can specifically block the adhesion of human cells to the human form of MMP-cleaved OPN and, furthermore, we show a successful active immunization strategy to specifically target human OPN thereby reducing its adhesive function *in vitro*. Further investigations are warranted to test whether active or passive immunization will also reduce inflammatory processes *in vivo*.

## Supporting Information

S1 Fig Quality controls of recombinant OPN. (A) Coomassie stain of recombinant OPN. (B) Immunoblot of recombinant OPN probed with polyclonal anti-OPN antibody (AF1433, R&D Systems).
(TIF)

S2 Fig Coating of OPN forms to microplates. Modified BCA protein assay of 10 μg/ml coated proteins. As a control 6 wells were incubated with coating buffer, the samples were coated overnight at 4°C in triplicates. * indicate a significant difference in comparison to all the other samples.
(TIF)

S1 Table List of Fluorescent antiboides used for FCm analysis. (DOCX)
