# Supplementary figures and images for: Inhibition of Cellular Adhesion by Immunological Targeting of Osteopontin Neoepitopes Generated through Matrix Metalloproteinase and Thrombin Cleavage
Source: PLoS One. 2016 Feb 3;11(2):e0148333. doi: 10.1371/journal.pone.0148333 (PMC4740464; doi:10.1371/journal.pone.0148333)

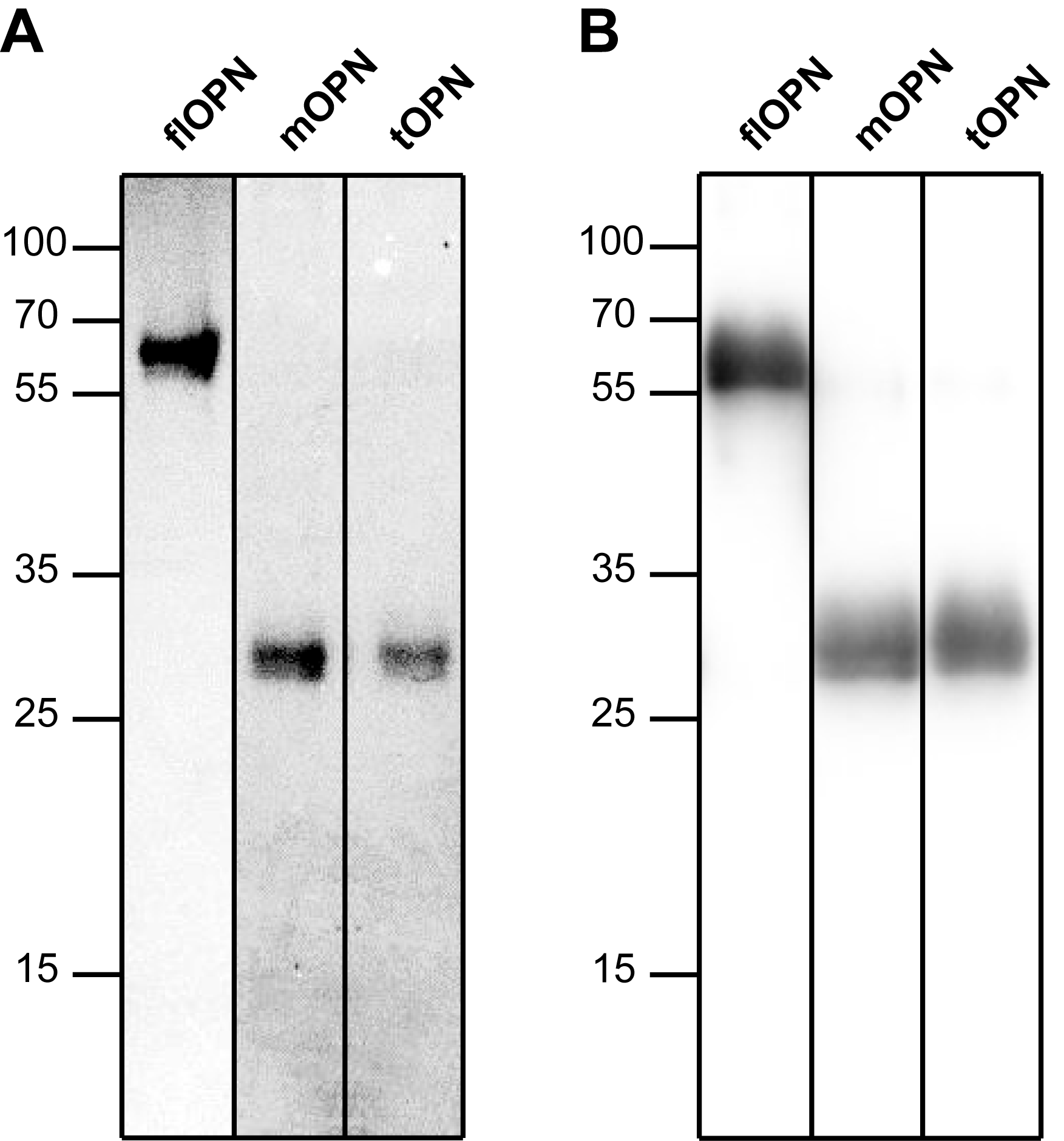

Supplement: S1 Fig — (A) Coomassie stain of recombinant OPN. (B) Immunoblot of recombinant OPN probed with polyclonal anti-OPN antibody (AF1433, R&D Systems). (TIF) [file pone.0148333.s001.tif]

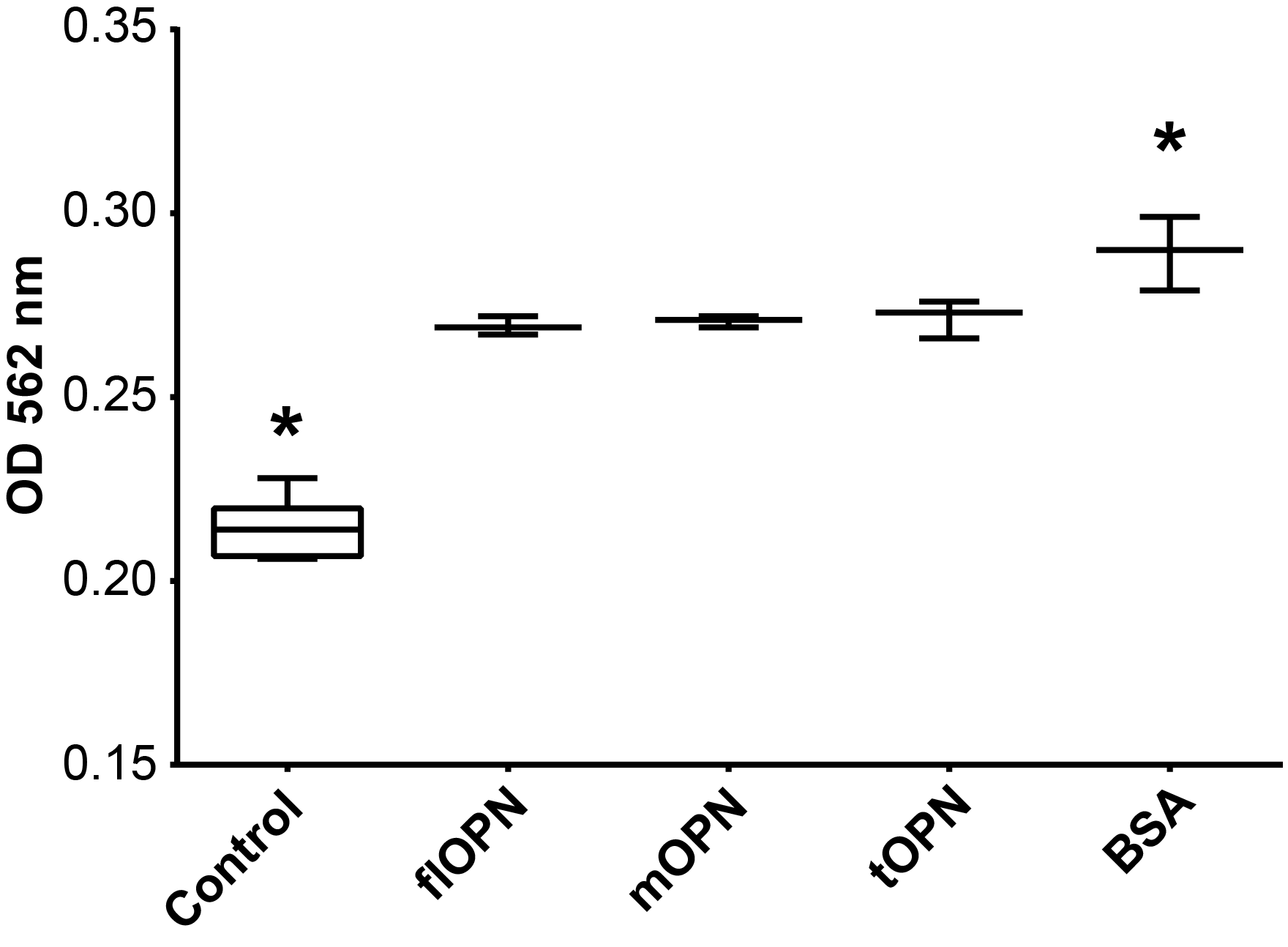

Supplement: S2 Fig — Modified BCA protein assay of 10 μg/ml coated proteins. As a control 6 wells were incubated with coating buffer, the samples were coated overnight at 4°C in triplicates. * indicate a significant difference in comparison to all the other samples. (TIF) [file pone.0148333.s002.tif]
