# Supplementary material for: Inhibition of Cellular Adhesion by Immunological Targeting of Osteopontin Neoepitopes Generated through Matrix Metalloproteinase and Thrombin Cleavage
Source: PLoS One. 2016 Feb 3;11(2):e0148333. doi: 10.1371/journal.pone.0148333 (PMC4740464; doi:10.1371/journal.pone.0148333)
Supplement: S1 Table — (DOCX) [file pone.0148333.s003.docx]

**Table 1** List of fluorescent antibodies used for FCM analysis

| **Integrin** | **Fluorescent-label** | **Clone** | **Company** |
| --- | --- | --- | --- |
| α_4_ | PE | 9F10 | eBioscience |
| α_5_ | PE | P106 | eBioscience |
| α_8_ | PE | 481709 | R&D Systems |
| α_v_ | PE | RMV-7 | eBioscience |
| α_9_β_1_ | PE | Y9A2 | BioLegend |
| β_1_ | FITC | T2/16 | eBioscience |
| β_3_ | FITC | VI-PL2 | eBioscience |
| β_5_ | FITC | KN52 | eBioscience |
| β_7_ | FITC | FIB504 | eBioscience |
| Control IgG | FITC | P3.6.2.8.1 | eBioscience |
| Control IgG | PE | 27-35 | BD Biosciences |
| CD34 | PerCP | 8G12 | BD Biosciences |
| CD45 | APC | 2D1 | BD Biosciences |
| CD144 | PE | 55-7H1 | BD Biosciences |
| CD144 | FITC | TEA 1/31 | Beckman Coulter |
